# Supplementary material for: The Small RNA Universe of Capitella teleta
Source: Front Mol Biosci. 2022 Feb 25;9:802814. doi: 10.3389/fmolb.2022.802814 (PMC8915122; doi:10.3389/fmolb.2022.802814)
Supplement: Supplementary file 1 [file DataSheet1.ZIP › Supplement/known/CAPTEscaffold_18_2262.pdf]

Provisional ID	:	CAPTEscaffold_18_2262
Score total	:	23322.5
Score for star read(s)	:	3.9
Score for read counts	:	23311.9
Score for mfe	:	2.1
Score for randfold	:	1.6
Score for cons. seed	:	3
Total read count	:	45737
Mature read count	:	44654
Loop read count	:	0
Star read count	:	1083

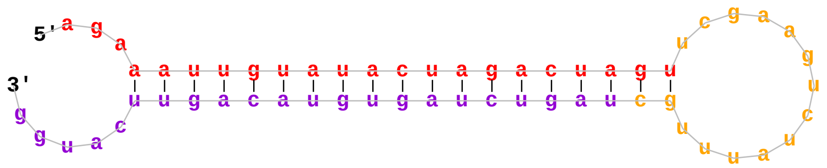

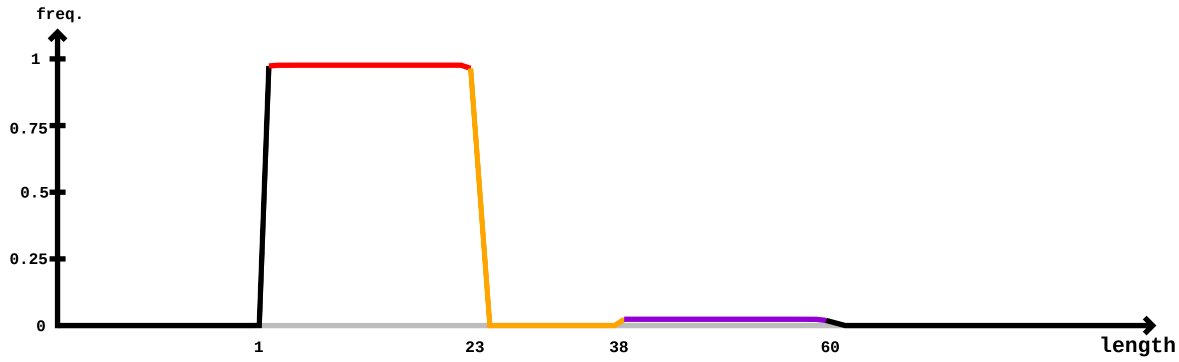

Mature **Star**

```
5'- cgacaucgcucgugggucucagaaauuguauacuagacuaguucgaagucuauuugcuagucuaguguacaguucaugggaucucagagcacugacuucuccgaaauggaca   -3'   obs
    cgacaucgcucgugggucucagaaauuguauacuagacuaguucgaagucuauuugcuagucuaguguacaguucaugggaucucagagcacugacuucuccgaaauggaca         exp
    cgacaucgcucgugggucucagaaauuguauacuagacuaguucgaagucuauuugcuagucuaguguacaguucaugggaucucagagcacugacuucuccgaaauggaca         known
    .......((((..((((((((..((((((((((((((((((............))))))))))))))))))..))))))))..)))).........(((.....)))..   reads   mm   sample

    ................Aucagaaauuguauacuagacuagu......................................................................   1     1   seq
    ..................Uagaaauuguauacuagacuagu.......................................................................   1     1   seq
    ..................cagaaauuguauacuagacuagu.......................................................................   5     0   seq
    ....................agaaauuguauacuagac..........................................................................   1     0   seq
    ....................agaaauuguauacuagacu.........................................................................   2     0   seq
    ....................agaaauAguauacuagacuag.......................................................................   1     1   seq
    ....................agaaauugAauacuagacuag.......................................................................   1     1   seq
    ....................agaaauuguauacuagacuaA.......................................................................   1     1   seq
    ....................agaaauuguauacuagacuag.......................................................................   535   0   seq
    ....................agaaUuuguauacuagacuag.......................................................................   1     1   seq
    ....................agaGauuguauacuagacuag.......................................................................   2     1   seq
    ....................agaaauuguauacuagacuaUu......................................................................   3     1   seq
    ....................agaaauuguauacuagGcuagu......................................................................   11    1   seq
    ....................agaaauugGauacuagacuagu......................................................................   3     1   seq
    ....................agaUauuguauacuagacuagu......................................................................   3     1   seq
    ....................agaaauuguauGcuagacuagu......................................................................   4     1   seq
    ....................aAaaauuguauacuagacuagu......................................................................   19    1   seq
    ....................agaaauuguauacuagacuagG......................................................................   9     1   seq
    ....................agaaauAguauacuagacuagu......................................................................   57    1   seq
    ....................agUaauuguauacuagacuagu......................................................................   5     1   seq
    ....................agaaauuguauacuagaAuagu......................................................................   11    1   seq
    ....................agaaauuguauacuaAacuagu......................................................................   12    1   seq
    ....................agaaUuuguauacuagacuagu......................................................................   2     1   seq
    ....................agaaauuguauacuagacuagC......................................................................   2     1   seq
    ....................agaaauuguauacuaCacuagu......................................................................   5     1   seq
    ....................agaaauuguauacuagUcuagu......................................................................   20    1   seq
    ....................agaaauuguauacAagacuagu......................................................................   15    1   seq
    ....................agaaauuguaGacuagacuagu......................................................................   3     1   seq
    ....................aNaaauuguauacuagacuagu......................................................................   1     1   seq
    ....................agaaauuguauaGuagacuagu......................................................................   5     1   seq
    ....................agaGauuguauacuagacuagu......................................................................   205   1   seq
```

```
                    Mature                                  Star
cgacaucgcucgugggucucagaaauuguauacuagacuaguucgaagucuauuugcuagucuaguguacaguucauggggaucucagagcacugacuucuccgaaauggaca
....................agaaauuguauacuaUacuagu......................................................................    5       1       seq
....................agaaauuguauaAuagacuagu......................................................................    11      1       seq
....................agaaauuguauacuGgacuagu......................................................................    16      1       seq
....................agaaauuguauaUuagacuagu......................................................................    1       1       seq
....................agaaauuguauacGagacuagu......................................................................    1       1       seq
....................agaaauuguauacuagacuGgu......................................................................    4       1       seq
....................Ggaaauuguauacuagacuagu......................................................................    11      1       seq
....................agaaauuguauacuagCcuagu......................................................................    3       1       seq
....................agaaauuguauacuagaUuagu......................................................................    1       1       seq
....................agaaauuguauacuagacuagu......................................................................    43101   0       seq
....................agaaauuguauacuagaGuagu......................................................................    2       1       seq
....................agaaauuguaCacuagacuagu......................................................................    5       1       seq
....................agaaauugAauacuagacuagu......................................................................    18      1       seq
....................agaaaCuguauacuagacuagu......................................................................    5       1       seq
....................agaaauuguauacuagacCagu......................................................................    4       1       seq
....................agaaauugCauacuagacuagu......................................................................    3       1       seq
....................Ugaaauuguauacuagacuagu......................................................................    4       1       seq
....................agaaauuguUuacuagacuagu......................................................................    2       1       seq
....................agaaauuguauacuagacuCgu......................................................................    1       1       seq
....................agaaauuguaAacuagacuagu......................................................................    28      1       seq
....................agaaauCguauacuagacuagu......................................................................    10      1       seq
....................agaaauuguauacuagacuagA......................................................................    33      1       seq
....................agaaauuguauacuUgacuagu......................................................................    4       1       seq
....................agaaauuguauacuagacAagu......................................................................    17      1       seq
....................agaaGuuguauacuagacuagu......................................................................    10      1       seq
....................agaaauuUuauacuagacuagu......................................................................    2       1       seq
....................agaaauuguauacuagacuaCu......................................................................    1       1       seq
....................Ngaaauuguauacuagacuagu......................................................................    9       1       seq
....................agaaauuCuauacuagacuagu......................................................................    4       1       seq
....................agaaauuguauUcuagacuagu......................................................................    13      1       seq
....................agaaaGuguauacuagacuagu......................................................................    3       1       seq
....................aUaaauuguauacuagacuagu......................................................................    5       1       seq
....................agaaauuguauacuagacGagu......................................................................    5       1       seq
....................agaaauGguauacuagacuagu......................................................................    5       1       seq
....................agaaauuguauacuagacuaAu......................................................................    1       1       seq
....................agaaauuAuauacuagacuagu......................................................................    10      1       seq
....................agGaauuguauacuagacuagu......................................................................    75      1       seq
....................agaaauuguauacuagacuUgu......................................................................    2       1       seq
....................aCaaauuguauacuagacuagu......................................................................    2       1       seq
....................agaaauuguGuacuagacuagu......................................................................    6       1       seq
....................agaaaAuguauacuagacuagu......................................................................    21      1       seq
....................agaaauuguauacCagacuagu......................................................................    5       1       seq
....................agaGauuguauacuagacuaguu.....................................................................    4       1       seq
....................agaaauuguauacuagacuaguu.....................................................................    101     0       seq
....................agaaauuguauacuagacuaguA.....................................................................    6       1       seq
....................agaaauuguauacuagacuaguN.....................................................................    1       1       seq
.....................gaaauuguauacuagacuagu......................................................................    108     0       seq
.....................gaaauuguauacuaUacuagu......................................................................    1       1       seq
.....................gaaauuguauaAuagacuagu......................................................................    1       1       seq
.....................gaaauuguauacuagacuaUu......................................................................    1       1       seq
.....................gaaauuguGuacuagacuagu......................................................................    2       1       seq
.....................gaaaAuguauacuagacuagu......................................................................    1       1       seq
.....................gaaauuguUuacuagacuagu......................................................................    3       1       seq
.....................gaaauuguauacuagaAuagu......................................................................    1       1       seq
......................aaauuguauacuagacuagu......................................................................    2       0       seq
.......................aauuguauacuagacuagu......................................................................    6       0       seq
........................auuguauacuagacuagu......................................................................    2       0       seq
.......................................................cuagucuaguguacaguucaug...................................    2       0       seq
.......................................................cuagucuagugAacaguucaugg..................................    1       1       seq
.......................................................cuagucuaguguacaguucaugg..................................    2       0       seq
........................................................uagucuaguguacaguuca.....................................    10      0       seq
........................................................uagucuaguguacagAucau....................................    1       1       seq
........................................................uagucuaguguacaguucau....................................    5       0       seq
........................................................uagucuaguguacaguucaug...................................    178     0       seq
........................................................uagucuaguguacaguucaAg...................................    1       1       seq
........................................................uagucuaguguacaguGcaug...................................    1       1       seq
........................................................uaAucuaguguacaguucaugg..................................    1       1       seq
........................................................uagucuaguguUcaguucaugg..................................    1       1       seq
........................................................uagucuaguCuacaguucaugg..................................    1       1       seq
........................................................uagucuaguguaGaguucaugg..................................    2       1       seq
```

Mature **Star**

```
cgacaucgcucgugggucucagaaauuguauacuagacuaguucgaagucuauuugcuagucuaguguacaguucauggggaucucagagcacugacuucuccgaaauggaca
.........................................................uagucuaguguacaguucauAg.................................    3    1    seq
.........................................................uagucuaguguacaguuUaugg.................................    2    1    seq
.........................................................Gagucuaguguacaguucaugg.................................    1    1    seq
.........................................................uagucuaguguacaguucaugg.................................    707  0    seq
.........................................................uGgucuaguguacaguucaugg.................................    1    1    seq
.........................................................uagucuaguguacaguucaAgg.................................    1    1    seq
.........................................................Nagucuaguguacaguucaugg.................................    2    1    seq
.........................................................uagucuGguguacaguucaugg.................................    1    1    seq
.........................................................uagucuaguguacaguGcaugg.................................    1    1    seq
.........................................................Aagucuaguguacaguucaugg.................................    4    1    seq
.........................................................uagucuaguguacaguucauggA................................    144  1    seq
.........................................................uagucuaguguacaguucauggU................................    9    1    seq
.........................................................uagucuaguguacaguucauggC................................    1    1    seq
```
